# Persons With Spinal Cord Injury Report Peripherally Dominant Serotonin-Like Syndrome After Use of Serotonergic Psychedelics

**DOI:** 10.1089/neur.2023.0022

**Published:** 2023-08-22

**Authors:** Stephanie Karzon Abrams, Brenden Samuel Rabinovitch, Rayyan Zafar, Aly Shah Aziz, Nicholas Paul Cherup, David W. McMillan, Jessica L. Nielson, Evan Cole Lewis

**Affiliations:** ^1^ Numinus Toronto, Toronto, Ontario, Canada.; ^2^ Centre for Psychedelic Research and Neuropsychopharmacology, Imperial College, London, United Kingdom.; ^3^ Pediatric Neurology Clinic, Oakville, Ontario, Canada.; ^4^ The Miami Project to Cure Paralysis, University of Miami Leonard M. Miller School of Medicine, Miami, Florida, USA.; ^5^ Department of Neurological Surgery, University of Miami Leonard M. Miller School of Medicine, Miami, Florida, USA.; ^6^ Department of Psychiatry and Behavioral Sciences; Institute for Health Informatics, University of Minnesota, Minneapolis, Minnesota, USA.; ^7^ Department of Pediatrics, Temerty Faculty of Medicine, University of Toronto, Toronto, Ontario, Canada.; ^8^ Division of Fundamental Neurobiology, Krembil Research Institute, Toronto, Ontario, Canada.; ^9^ Department of Physiology, Temerty Faculty of Medicine, University of Toronto, Toronto, Ontario, Canada.

**Keywords:** LSD, MDMA, psilocybin, psychedelics, serotonin, spasms

## Abstract

Psychedelic-assisted therapy (PAT) may treat various mental health conditions. Despite its promising therapeutic signal across mental health outcomes, less attention is paid on its potential to provide therapeutic benefits across complex medical situations within rehabilitation medicine. Persons with spinal cord injury (SCI) have a high prevalence of treatment-resistant mental health comorbidities that compound the extent of their physical disability. Reports from online discussion forums suggest that those living with SCI are using psychedelics, though the motivation for their use is unknown. These anecdotal reports describe a consistent phenomenon of neuromuscular and autonomic hypersensitivity to classical serotonergic psychedelics, such as psilocybin and lysergic acid diethylamide (LSD). Persons describe intense muscle spasms, sweating, and tremors, with an eventual return to baseline and no reports of worsening of their baseline neurological deficits. The discomfort experienced interferes with the subjective beneficial effects self-reported. This phenomenon has not been described previously in the academic literature. We aim to provide a descriptive review and explanatory theoretical framework hypothesizing this phenomenon as a peripherally dominant serotonin syndrome-like clinical picture—that should be considered as such when persons with SCI are exposed to classical psychedelics. Raising awareness of this syndrome may help our mechanistic understanding of serotonergic psychedelics and stimulate development of treatment protocols permitting persons with SCI to safely tolerate their adverse effects. As PAT transitions from research trials into accepted clinical and decriminalized use, efforts must be made from a harm reduction perspective to understand these adverse events, while also serving as an informed consent process aid if such therapeutic approaches are to be considered for use in persons living with SCI.

## Introduction

Psychedelic-assisted therapy (PAT) has demonstrated success in treating a variety of mental health conditions, including major depressive disorder (MDD),^1^ treatment-resistant depression (TRD),^2^ substance use disorder,^3,4^ post-traumatic stress disorder (PTSD),^5,6^ and existential anxiety at end of life.^6,7^ The U.S. Food and Drug Administration has designated 3,4-methylenedioxy-methamphetamine (MDMA) and psilocybin—one of the psychoactive components in “magic” mushrooms—as breakthrough therapies for PTSD and MDD, respectively. Since January 2022 in Canada, physicians have been able to request MDMA and psilocybin for patients with “serious or life-threatening conditions” by application to the Special Access Program.^2^ In February 2023, the Therapeutic Goods Administration of Australia announced that by July 2023, qualified psychiatrists will be able to prescribe MDMA for PTSD and psilocybin for TRD.^8,9^

To date, PAT has avoided or actively excluded persons with chronic or serious “medically significant” cardiovascular and autonomic status as well as those with specific neurological diagnoses.^10,11^ This has included persons with spinal cord injury (SCI), who have a high prevalence of treatment-resistant mental health comorbidities.^12–17^ Anecdotal reports document these persons using illegally sourced psychedelics, without clinical supervision, resulting in an absence of published academic literature on the topic.^18,19^

The World Health Organization estimates a global prevalence of 250,000–500,000 persons living with a SCI.^20^ These persons have a high burden of mental health disorders, such as depression and anxiety.^21–24^ To date, there are no clinical data to guide psychedelic practitioners and therapists for persons with SCI requesting PAT. In the absence of clinical guidance and supervision, self-treatment could lead to a greater likelihood of psychological and physical adverse events.

According to online reporting, self-guided psychedelic exploration occurs in the SCI population, with many describing a worrisome set of adverse events. Posts on the freely accessible internet forum sites Reddit and Shroomery describe accounts of a common phenomenon experienced after ingestion of serotonergic psychedelic substances, such as psilocybin and lysergic acid diethylamide (LSD). They describe a constellation of symptoms marked by intense, non-ordinary, and uncomfortable leg spasms.^18,19^ Given that these specific symptoms are not reported by those without SCI, this phenomenon appears to be unique to this population, perhaps signaling a specific physiological response that warrants further investigation. It highlights important questions about the neuropsychopharmacology of psychedelic medicines and the underlying mechanisms of spasticity in SCI.

## Methods

To find the online posts describing the phenomena we report here, a comprehensive search was done on the forum websites Reddit and Shroomery. The search function on both of these websites was used to find subforums and posts pertaining to SCI and psychedelic use. An initial search was done to find the subforums with posts discussing these topics. The keywords used were “psychedelics,” “spinal cord injury,” “SCI,” “magic mushrooms,” “psilocybin,” “LSD,” “serotonergic,” “spasms,” “muscle jerks,” “paralysis,” and “paralyzed.” All of the user posts and comments were screened by their title and content to ensure that they contained descriptions of the type of SCI and serotonergic psychedelic use. Posts that did not contain both the SCI and serotonergic psychedelic descriptions were not included in the analysis. Once the final batch of posts were approved for analysis for this study, the key information was extracted, regarding the psychedelic use and reported effects. Experiences reported by the online users were then matched with the respective clinical symptomatology.

## Rationale for Adverse Events in Persons Living With Spinal Cord Injury Who Take Serotonergic Psychedelics

### 5HT2A receptor

The 5HT2A receptor (5HT2A-R) is abundant within the central nervous system, most notably in brain regions implicated in mental health disorders, cognitive processing, and on the interneurons and motor neurons of the spinal cord facilitating sensorimotor function.^25^ The 5HT2A-R also plays an important role in the regulation of skeletal muscle tone through its action at neuromuscular junctions.^26–28^ Peripherally, the 5HT2A-R is found in the gastrointestinal tract, vascular smooth muscle, bronchial smooth muscle, and platelets.^29,30^

### Psychedelics and 5HT2A-R

Classical psychedelics have direct agonism at the 5HT2A-R. These include psilocybin-containing mushrooms (e.g., “magic mushrooms,” “shrooms,” and “mushies”), LSD (“acid”), mescaline-containing cacti (e.g., “Peyote,” “San Pedro,” and “buttons”), and dimethyltryptamine (e.g., N,N-DMT, 5-MeO-DMT). MDMA is an atypical psychedelic referred to as an empathogen or entactogen because of its effects and mechanism. Although not a classical psychedelic, it does provide indirect activation of the 5HT2A-R by increasing serotonin concentrations within synaptic clefts through multiple mechanisms.^31^ Other substances culturally identified with a “psychedelic” monomer have varying serotonergic and 5HT2A-R effects (e.g., ketamine).

In clinical trials, therapeutic effects of classical psychedelics in the treatment of mental health conditions have been postulated to occur, in part, because of the agonism of the 5HT2A-R in the brain.^6,32^ However, given that psychedelics also act on the 5HT2A-R within the spinal cord, this may explain inadvertent physiological effects reported, which include shaking/trembling,^33^ nausea,^33,34^ discomfort,^33^ increased heart rate,^33^ and disorientation.^34^ The constellation of these unwanted symptoms and signs are similar in description, but not in severity, to what is experienced in conditions of significant serotoninergic excess, such as serotonin syndrome (SS).^35–37^

### Serotonin syndrome

SS is a serious, life-threatening condition that presents with mental status changes, autonomic system hyperactivity, and neuromuscular abnormalities (Table 1). Its mechanism involves increased synaptic serotonin (5-hydroxytryptamine; 5-HT) concentration or direct activation of 5-HT receptors. Both the 5HT1A and 5HT2A receptor subtypes are implicated in producing the clinical manifestations of the syndrome in both humans and mouse models.(54,55) SS can be precipitated by increased exposure to or overdoses with serotonergic drugs (e.g., selective serotonin reuptake inhibitors or serotonin and norepinephrine reuptake inhibitors) or the combination of serotonergic drugs with medications that prevent its breakdown, such as monoamine oxidase inhibitors.^35,38–41^

**Table 1.** Signs and Symptoms of Serotonin Syndrome

Symptom cluster	Symptomatology
Altered mental status	Agitation Anxiety Disorientation Restlessness Excitement
Neuromuscular abnormalities	Tremors Clonus Hyperreflexia Muscle rigidity Bilateral Babinski signs Akathisia
Autonomic hyperactivity	Hypertension Tachycardia Tachypnea Hyperthermia Mydriasis Diaphoresis Dry mucous membranes Flushed skin Shivering Vomiting Diarrhea Increased bowel sounds Arrhythmias

Table adapted from Maitland and colleagues (2022), Wang and colleagues (2016), Francescangeli and colleagues (2019), Foong and colleagues (2018), and Tormoehlen and colleagues (2018).

### Description of the experience of persons with spinal cord injury with serotonergic psychedelics

Persons with SCI are using online social media platforms, such as Reddit and Shroomery, to document their psychedelic experiences outside of supervised clinical settings.^18,19^ Table 2 summarizes an example of descriptions of these experiences and details the corresponding clinical symptomatology and psychedelic compounds utilized.

**Table 2.** Excerpts From Discussion Forums and Corresponding Clinical Symptomatology

Data source	SCI level	Psychedelic used	User description of experience	Clinical symptomatology
Reddit	T8: complete	Not specified	**Psychedelic User 1** “I've been paraplegic for 25 years (T8 complete).…what I got was absolutely no psychoactive effect whatsoever and leg spasms so bad I couldn't get up from my seat for several hours; the spasming relaxed a bit but they continued for the next 2–3 days so that what is usually a minor issue for me made life difficult.” r/shrooms Posted by u/Special_Ed2018 2020 Spinal cord injury and shrooms	Lower limbs: muscle spasm Upper limbs: not reported Additional: not reported
Reddit	T10: complete	Psilocybin mushrooms (5 g, dried)	**Psychedelic User 2** “T10 complete here. I get spasms too. It helps to cross my legs in a meditative fashion, 5+ grams silent darkness. “Heroic Dose”, only way to do it in my opinion. I personally don't take any of the muscle relaxers or lyrica stuff I was prescribed. I eventually got used to being without it. My doctor worked with me and prescribes Diazepam for acute spasms and that helps on the come down. On the come up my feet lock into place, toe down and if I try to move them to get in my chair or something I look almost convulsive.” r/shrooms Posted by u/MaybeMushy 2020 Spinal cord injury and shrooms	Lower limbs: muscle spasm; cramp/contracture; clonus Upper limbs: not reported Additional: not reported
Reddit	Not specified	LSD (100 μg)	**Psychedelic User 3 “**I became paraplegic 3 years ago and during that time I experimented with LSD once, that moment I took half a dose, approximately 100 μg and a few hours later I was sweating cold, I have spasms but they were never as serious as that time where my legs did not stop move, apart from that the trip was interesting but quite annoying about the spasms.” r/lsd Posted by u/EIM4cH027 2020 LSD and spinal cord injury	Lower limbs: muscle spasm Upper limbs: not reported Additional: diaphoresis; temperature dysregulation (cold)
Reddit	T4: incomplete	Multiple experiences, drug not specified	**Psychedelic User 4** “Initially I was a T4 injury. I have some control below my original nipple line. Within 30 minutes of taking any psychedelic my legs begin jumping in my chair like in nervous. The muscles up to my original level of injury begin tightening and contracting. I become dehydrated. I feel like I'm floating on top my chair rather than in it. And I go through periods of extreme warm to comfortable.” r/psychedelics Posted by u/JesiStracham 2020 Spinal cord injury and psychedelics—mushrooms, acid, MDMA, X	Lower limbs: muscle spasms; cramp/contracture Upper limbs: not reported Additional: temperature dysregulation (cold); muscle cramp/contracture in abdomen and torso
Reddit	C5/6: incomplete	Not specified	**Psychedelic User 5** “I have a minor SCI at C5–C6, intermittently my left leg spasms, has clonus, or cramps and locks up. When I take psychedelics now I get a sharp pain, cramping, and spasms in my right leg which normally doesn't experience these sort of issues.” r/shrooms Posted by u/otishotpie 2022 Spinal cord injury and shrooms	Lower limbs (right-side): muscle spasms; cramp/contracture; pain Upper limbs: not reported Additional: not reported
Reddit	T10: not specified	Psilocybin mushrooms (3 g, dried)	**Psychedelic User 6** “t-10 paraplegic here I did about 3g of mushrooms and same with my legs jumping and spasms for about 3hr not the finest part but I did enjoy it. Now I think I might start to micro dose I have heard of people getting some feeling and/or bowl or bladder function back so its worth a shot to try it. I don't take any prescribed drugs any more it would make me sleepy and cloud my thoughts.” r/shrooms Posted by u/mpancho167 2020 Spinal cord injury and shrooms	Lower limbs: muscle spasms Upper limbs: not reported Additional: not reported
Shroomery	Not specified	Hallucinogens (not specified)	**Psychedelic User 7** **“**So I was in an accident several years back and injured my spine. Since I have been injured any type of hallucinogenic causes me to have very strong muscle spasms followed by sweating profusely, heightened blood pressure and heart rate.” Mushrooms, Mycology and Psychedelics Posted by iloveshitaki, #14799884 2022 The psychedelic experience	Lower limbs: muscle spasms (location not reported) Upper limbs: muscle spasms (location not reported) Additional: diaphoresis; tachycardia; increased blood pressure

SCI, spinal cord injury; LSD, lysergic acid diethylamide; MDMA, 3,4-methylenedioxy-methamphetamine.

Several reports include SCI localization ranging from C4 from T10, and the type of psychedelics taken. Reported experiences include intense leg spasms, sensations of feet feeling immobile and locked, convulsions during attempts at motor movement, legs “jumping,” dehydration, cold sweats, overheating, sensations of extreme heat, urinary incontinence, and sharp pains in the spasming legs. One online conversation (user 10 and user 11) discussed pre-medicating with baclofen or diazepam to prevent spasms. Reports consistently note an extreme severity of symptoms and how this, at times, acts as a barrier to safe use. However, despite anticipated negative experiences and potential harms, some reports describe forgoing the risk and committing to using a psychedelic compound. For example, a report from a person with an incomplete T10 lesion (user 12), notes that psilocybin “changed his life” with respect to his PTSD.

## Rationale for Peripheral Serotonin-Like Syndrome as a Mechanism for Adverse Events of Psychedelic Use in Persons Living With Spinal Cord Injury

### Proposed mechanism for the described clinical effects

There appears to be shared clinical features between SS and reported adverse effects of psychedelics in those with SCI. This population-specific manifestation warrants closer scrutiny given the low risk of physiological toxicity in persons without SCI.^42^ Knowing the mechanism of SS, the use of serotonergic psychedelic substances in persons with SCI suggests an induced peripherally dominant SS-like clinical picture.

Post-SCI, a series of anatomical and functional changes occur in the spinal cord, including reorganization of the spinal neuronal network, alteration of properties of interneurons, and motor neurons as well as up- or downregulation of different neurotransmitter receptors. Specifically, during complete spinal cord transection, the descending supply of 5-HT is lost. As a consequence, 5-HT receptors undergo variant degrees of expression, which can be modulated by 5-HT signaling.^26^ The descending pathways include direct motor-initiating pathways, such as corticospinal tracts, and modulatory pathways, such as serotonergic, dopaminergic, glutamatergic, and noradrenergic pathways. In animal models, these monoaminergic systems exert significant effects in both acute or chronic SCI, given that direct stimulation of receptors by these monoamines with synthetics can regain locomotor activity.^43,44^ Concurrent 5HT2/α1 agonism acutely increased motor neuron excitability in adults with chronic SCI,^45^ pointing toward the neuromotor potential of these substances. In this study, induced spasticity was also increased, similar to what has been noted by those self-administering psychedelics in non-laboratory settings.

The peripheral effects of serotonin occur in the short and long term. Short-term immediate effects modulate skeletal muscle tone, whereas longer-term effects involve intracellular second messenger signaling, depending on the serotonin receptor subtype. A decrease in serotonergic activity attributable to SCI may lead to chronic upregulation in the expression of serotonin receptor subunit-encoding genes in the periphery, resulting in increased surface expression of 5HT2A-R and a state of peripheral hypersensitivity to serotonergic compounds with preference for this receptor. Thus, exposure to 5HT2A-R agonists, such as the classical psychedelics, could result in differential peripheral effects between SCI and non-SCI populations without a notable difference in the central (psychological) experiences reported in the literature.

From the online posts, this appears to be the case given that persons with SCI are describing similar degrees of psychological benefit while reporting differential peripheral experiences (Table 2). At common, standard doses, an unexpected peripherally dominant SS-like clinical picture, associated with expected central effects, appears to manifest in this population.

Pre-clinical studies could be conducted that directly test the impact of 5HT2A-R psychedelic agonists in animal models of SCI. These studies should aim to establish: 1) changes in 5HT2A-R availability in the spinal cord post-administration of psychedelics; 2) changes in serotonergic neurotransmission both acutely and subacutely in response to psychedelics; and 3) how such interactions impact core symptoms of SCI.

A last important point to consider is the incidence of autonomic dysreflexia (AD) in persons living with SCI. AD predominantly occurs in those with an injury at T6 or above, where trivial autonomic stimuli cause life-threatening increases in blood pressure and heart rate.^46^ Expected side effects of classical psychedelic use are safe, but clinically significant increases in blood pressure and heart rate may occur.^47^ As such, those with uncontrolled hypertension, or cardiovascular disease, are often excluded for safety. Given that one of the common physical symptoms of AD is excessive sweating/hyperthermia, and some reports from the discussion forums reported this reaction, persons with injuries higher on their spinal cord may be more prone to AD and caution should be taken when considering the use of classical psychedelics in this population.

## Conclusion

Our article provides an account of the reported experience of autonomic and neuromuscular hyperactivity, underscored by intense muscle spasms, that is consistently reported by persons with SCI in the context of serotonergic psychedelic use. We also postulate a mechanism of this phenomenon. Characterization and severity of these symptoms have not been reported in published clinical psychedelic medicine trials with use of similar compounds at similar doses in the non-SCI population. The differential peripheral symptoms observed warrants further investigation. Our intent is to lay the foundation where a planned follow-up survey study in SCI patents will report on the prevalence and further specify clinical details of this novel phenomenon.

From online self-reports, it is clear that those with SCI are already exploring psychedelics despite uncomfortable adverse effects. This public commentary raises awareness of this phenomenon in the spirit of harm reduction and is a call to action to explore potential SCI-specific mechanism(s). A greater understanding will help develop a framework of SCI-specific considerations to guide clinicians and therapists for safe and effective use of psychedelics in this population, much like the patient-centered models that were originally established for primary PTSD, MDD, and other mental health conditions.

Additionally, exploration of such mechanism(s) will lead to improving our understanding of the pathophysiology of muscle spasms in SCI, thus promoting use of pharmacological interventions to reduce undesired spasms for persons with SCI choosing to use psychedelics.

## Abbreviations Used

5-HT: 5-hydroxytryptamine
5HT2A-R: 5HT2A receptor
AD: autonomic dysreflexia
LSD: lysergic acid diethylamide
MDD: major depressive disorder
MDMA: 3,4-methylenedioxy-methamphetamine
PAT: psychedelic-assisted therapy
PTSD: post-traumatic stress disorder
SCI: spinal cord injury
SS: serotonin syndrome
TRD: treatment-resistant depression
